# Influencing Factors and Adaptation Strategies of Stoichiometric Characteristics of Main Shrubs and Herbs in Karst Area at Microhabitat Scale

**DOI:** 10.3390/plants14182839

**Published:** 2025-09-11

**Authors:** Peng Wu, Hua Zhou, Wenjun Zhao, Guangneng Yang, Yingchun Cui, Yiju Hou, Chengjiang Tan, Ting Zhou, Run Liu, Fangjun Ding

**Affiliations:** 1 Guizhou Libo Karst Forest Ecosystem Observation and Research Station, Guizhou Academy of Forestry, Guiyang 550005, China; zuishaoxu@163.com (P.W.);; 2 Key Laboratory of National Forestry and Grassland Administration on Biodiversity Conservation in Karst Mountainous Areas of Southwestern China, Guizhou Academy of Forestry, Guiyang 550005, China; 3 Guizhou Liping Rocky Desertification Ecosystem Observation and Research Station, Guizhou Academy of Forestry, Guiyang 550005, China; 4 Maolan National Nature Reserve Administration, Libo 558400, China; 5 Guiyang Forest Chief Scheme Work Service Center, Guiyang 550002, China

**Keywords:** microhabitat, stoichiometric characteristics, impact factors, adaptive strategy, shrubs and herbs, Maolan National Nature Reserve

## Abstract

In order to reveal the adaptation strategies of karst forest plants to “high-calcium (Ca)–low-phosphorus (P) heterogeneous” habitats, the dominant shrubs and herbs in the Maolan karst area were taken as the research objects. The carbon (C), nitrogen (N), P, potassium (K), Ca, and magnesium (Mg) contents of plant components and their stoichiometric ratios in different microhabitats were systematically measured, and the environmental driving factors were analyzed by redundancy analysis (RDA) and variance partitioning analysis (VPA). The results showed that there were no significant differences in the plant nutrient contents and stoichiometric ratios in different microhabitats, but there were significant differences with respect to the components. The contents of N, P, K, and Mg in shrub leaves were significantly higher than those in branches and roots, while the contents of C/N, C/P, and C/K in branches and roots were significantly higher than those in leaves. The K content of herb leaves was significantly higher than that of roots. This reflects the functional differentiation of plant components and the different trade-off strategies for resource acquisition and storage. The stoichiometric characteristics of shrub leaves are dominated by species characteristics, while herb leaves are controlled by leaf tissue density (*LTD*), and soil-exchangeable Ca has a significant regulatory effect on the roots of both plant forms. Shrubs directly obtain bedrock slow-release nutrients through deep roots penetrating rock crevices and combine high C/N and C/P to improve nutrient utilization efficiency, forming a “mechanical resistance priority–metabolic cost optimization” adaptation strategy. Herbs respond to environmental fluctuations through functional trait plasticity and achieve rapid growth with high specific leaf area (*SLA*) and low *LTD*.

## 1. Introduction

Ecological stoichiometry is an interdisciplinary science that studies the energy balance and chemical element balance of biological systems [1]. It is closely related to plant individual growth and development, population dynamics, community species diversity, and ecosystem types [2]. It not only reflects the soil nutrient status and plant resource utilization efficiency and ecological adaptation strategy [3]; it also affects the function and succession of the whole ecosystem. The content of C, N, P, and K in leaves is the basic component of plant stoichiometry, and fluctuations thereof significantly affect the productivity of a forest ecosystem and changes in carbon sources and sinks [4]. Stoichiometric ratios are key for diagnosing nutrient limitations, understanding nutrient cycling [5], and evaluating plant adaptation to environmental changes [6]; at the same time, these stoichiometric characteristics can also be used as key indicators for determining the structure and function of the community and effects of the stability of the ecosystem [7].

As the largest, the most unique, and a relatively stable ecosystem in the same latitude zone of the world, Maolan karst forests, with their environmental particularity, structural complexity, system vulnerability, and restoration difficulty, constitute the key area of global biodiversity conservation [8]. As an important part of the ecosystem, shrubs and herbs have irreplaceable ecological value in maintaining this area’s stability, functionality, and sustainability [9]. They drive material circulation and energy flow through efficient photosynthesis. At the same time, under the particular environment in the karst area, with its shallow and uneven distribution of soil, its developed root network can effectively stabilize soil, prevent erosion, and significantly reduce soil erosion. In addition, these plants provide valuable habitats and abundant food resources for many animals and constitute the cornerstone for maintaining the biodiversity of karst forests [10]. More importantly, these plants have significantly enhanced the ecosystem’s ability to resist, and recover from, climate change and human disturbance through long-term adaptation to the complex and changeable environment in the karst area.

At the macro scale, climatic factors, especially heat and water conditions, as the dominant factors, shape the zonal distribution of vegetation types. At the landscape and even smaller scales, non-zonal environmental factors (geomorphological morphology, hydrological structure, etc.) are the main controlling factors driving the heterogeneity pattern of vegetation [11]. The surface bedrock in the karst area has a large area, and the terrain is undulating and changeable. The unique dual structure of ground–groundwater hydrology is superimposed to jointly construct a highly heterogeneous geomorphologic landscape. This special landform has given birth to a variety of microhabitat forms and their spatial combination types, including stone surface, stone gully, stone fissure, stone cave, stone trough, and soil surface [8], and different microhabitat types have shown significant spatial differentiation characteristics in the allocation of ecological elements. The soil showed a typical patchy discontinuous distribution pattern, and the accumulation of litter and the content of mineral elements showed a gradient change. The recharge–storage–evaporation process of soil moisture showed obvious spatial and temporal heterogeneity due to the existence of a hydrological dual structure. The spatial variation of these ecological factors not only directly determines the growth trend and community diversity level of plants by regulating key ecological parameters such as light intensity, water supply, and nutrient availability [12]; it also profoundly affects the adaptation strategy, spatial distribution pattern, and community succession process of plants to heterogeneous habitats [13].

The current research on plant stoichiometry focuses on the dominant tree species in forest ecosystems in specific regions. Jing et al. systematically studied the stoichiometric characteristics of six dominant tree species (*Betula platyphylla*, *Pinus armandii*, *Larix principis-rupprechtii*, *Quercus wutaishanica*, *Pinus tabulaeformis*, and *Populus davidiana*) in the semi-arid region of the Loess Plateau [14]. Dong et al. studied the seasonal variation of the stoichiometric characteristics of *Robinia pseudoacacia* in the hilly region of the Loess Plateau [15]. Our team also conducted a detailed analysis of the stoichiometric characteristics and influencing factors of 14 dominant tree species in the Maolan karst forests [16]. In contrast, the research on the stoichiometric characteristics of shrubs or herbs is still insufficient, especially in special areas such as karst areas, where the terrain is highly fragmented and the habitat heterogeneity is extremely strong. The extremely uneven distribution of resources influences the impact of environmental factors (such as topography, soil, and plant characteristics, etc.) and their interactions on the stoichiometric characteristics of plants, and how these plants adapt to habitat changes is still a question to be answered. In addition, existing studies are mostly limited to the analysis of the independent effects of a single environmental factor or a single plant functional trait; there is a lack of systematic discussion with respect to the synergistic effects of multiple factors and their comprehensive ecological effects.

Therefore, based on the Maolan karst forest ecosystem, this study takes the dominant shrubs and herbs at the microhabitat scale as the research object and systematically analyzes different components (shrubs: leaves, branches, and roots; herbs: leaves and roots). The contents of C, N, P, K, Ca, and Mg and their stoichiometric ratios (C/N, C/P, C/K, N/P, N/K, K/P, and Ca/Mg) are investigated. The following key scientific questions are explored: (1) Do shrubs and herbs differ significantly in element contents and stoichiometric ratios across microhabitats? (2) What is the internal coupling relationship between element content and stoichiometric ratio of plant components? (3) How do topographic factors, soil properties, and plant characteristics synergistically affect the stoichiometric characteristics of these plants? (4) How do shrubs and herbs adapt to microhabitat changes by regulating their own physiological and ecological processes? The results of this study will further elucidate the survival strategies of plants in harsh habitats and the mechanism of their adaptation to complex terrain and provide a theoretical basis for understanding the maintenance of karst forest ecosystem stability and the restoration of degraded karst forests under complex terrain conditions.

## 2. Results

### 2.1. Nutrient Contents of Different Components of Main Shrubs and Herbs in Karst Forests

#### 2.1.1. Nutrient Contents of Various Components of Main Shrub Plants in Karst Forests Under Different Microhabitats

**Figure 1:**
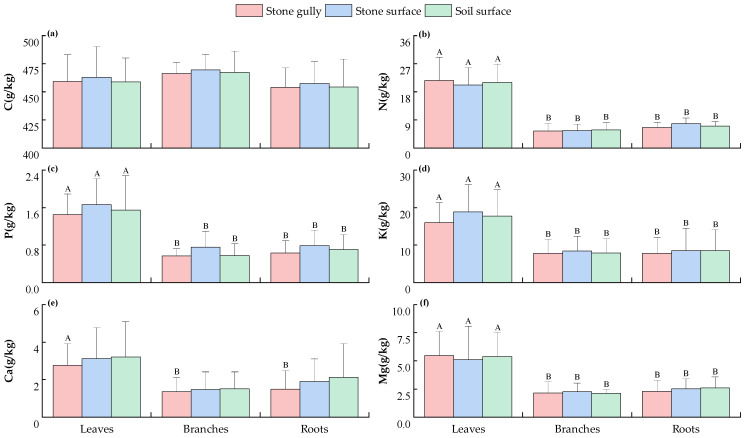
The contents of C (**a**), N (**b**), P (**c**), K (**d**), Ca (**e**), and Mg (**f**) of various components of the main shrub plants in karst forests under different microhabitats. Different capital letters indicate significant differences among different components of plants in the same microhabitat (*p* < 0.05). The absence of any letter indicates that there is no significant difference between different plant components or different microhabitats (*p* > 0.05).

The contents of C, N, P, K, Ca, and Mg in each component (leaf, branch, and root) of the dominant shrubs in karst forests under three typical microhabitats (stone gully, stone surface, and soil surface) were systematically analyzed (Figure 1). The results showed that under the same microhabitat conditions, the contents of N, P, K, and Mg in plant leaves were significantly higher than those in branches and roots (*p* < 0.05), while no significant differences were observed between branches and roots (*p* > 0.05). In addition, in the stone gully microhabitat, the Ca content in leaves was also significantly higher than that in branches and roots (*p* < 0.05), but in the stone surface and soil surface microhabitats, the differences in Ca content among different components was not significant (*p* > 0.05). It is worth noting that the distribution of C content as a structural element between different components showed a high degree of uniformity, and the difference was not significant (*p* > 0.05), which confirms its stability as a structural element.

Among different microhabitats, the contents of C, P, and K in each shrub component were the highest in the stone surface microhabitat, while the content of Ca was the highest in the soil surface microhabitat, but the differences between these elements in different microhabitats we not significant (*p* > 0.05). However, this trend still reflected the influence of karst environmental heterogeneity on nutrient distribution: in the stone surface microhabitat, due to the thin substrate and poor water retention, plants may enhance the photosynthetic metabolism by increasing the content of C, P, and K in leaves, while in the soil surface microhabitat, where soil development was relatively complete, the absorption of Ca by plants may be more dependent on the interaction between roots and soil. In addition, sample size limitations or environmental fluctuations (i.e., local moisture differences caused by microhabitats) may also affect statistical power. This trend still needs to be verified in subsequent studies with larger sample sizes and through multivariate analysis.

#### 2.1.2. Nutrient Contents of Various Components of the Main Herb Plants in Karst Forests Under Different Microhabitats

**Figure 2:**
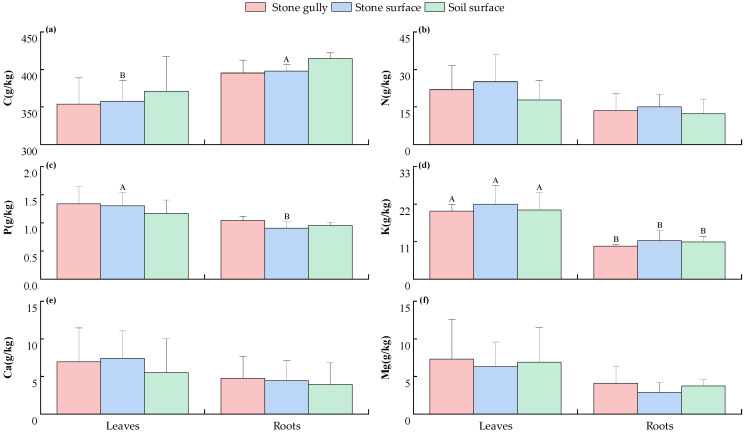
The contents of C (**a**), N (**b**), P (**c**), K (**d**), Ca (**e**), and Mg (**f**) of various components of the main herb plants in karst forests under different microhabitats. Different capital letters indicate significant differences among different components of plants in the same microhabitat (*p* < 0.05). The absence of any letter indicates that there is no significant difference between different plant components or different microhabitats (*p* > 0.05).

As shown in Figure 2, the element content of each component of the dominant herbs in karst forests shows the following distribution characteristics under different microhabitats. The C content of leaves and roots was the highest in the soil surface microhabitat, the N and K contents were the highest in the stone surface microhabitat, and the P and Mg contents were the highest in the stone gully microhabitat, but the differences in these elements in different microhabitats were not significant (*p* > 0.05). Under the same microhabitat conditions, the K content of plant leaves was significantly higher than that of roots (*p* < 0.05); in the stone surface microhabitat, the P content in leaves was also significantly higher than that in roots (*p* < 0.05), but the C content in roots was significantly higher than that in leaves (*p* < 0.05), while the distribution of N, Ca, and Mg contents in leaves and roots showed a high degree of uniformity, and the difference was not significant (*p* > 0.05).

### 2.2. Stoichiometric Characteristics of Different Components of Main Shrubs and Herbs in Karst Forests

#### 2.2.1. Stoichiometric Characteristics of Various Components of Main Shrub Plants in Karst Forests Under Different Microhabitats

**Figure 3:**
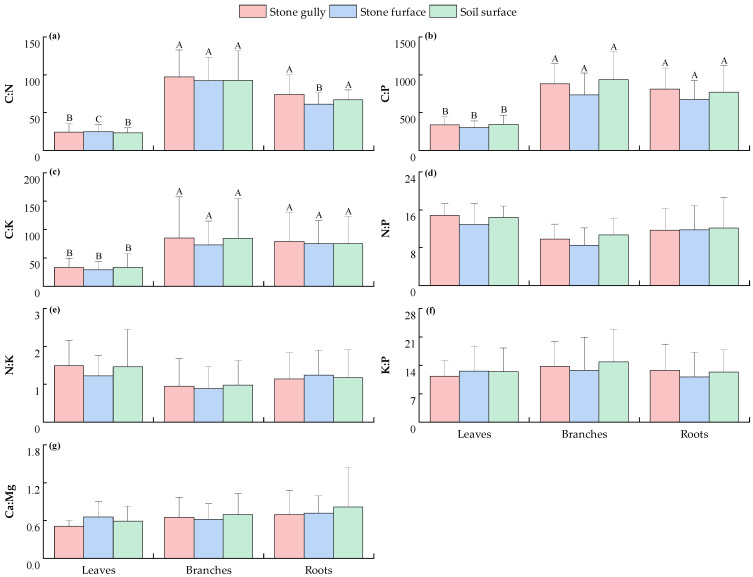
The C/N (**a**), C/P (**b**), C/K (**c**), N/P (**d**), N/K (**e**), K/P (**f**), and Ca/Mg (**g**) of various components of the main shrub plants in karst forests under different microhabitats. Different capital letters indicate significant differences among different components of plants in the same microhabitat (*p* < 0.05). The absence of any letter indicates that there is no significant difference between different plant components or different microhabitats (*p* > 0.05).

As shown in Figure 3, the stoichiometric ratios of leaves, branches, and roots of the dominant shrubs in karst forests showed no significant differences across the different microhabitats (*p* > 0.05). Further analysis showed that the C/N, C/P, and C/K of branches and roots were significantly higher than those of leaves under the same microhabitat conditions (*p* < 0.05). In addition, this study also found that the distribution of N/P, N/K, K/P, and Ca/Mg in different plant components showed a high degree of consistency, and the differences were not significant (*p* > 0.05).

#### 2.2.2. Stoichiometric Characteristics of Various Components of the Main Herb Plants in Karst Forests Under Different Microhabitats

**Figure 4:**
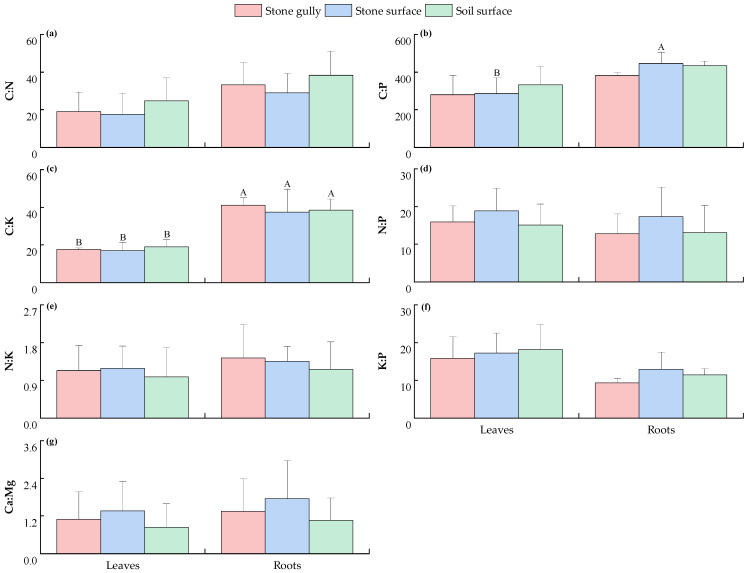
The C/N (**a**), C/P (**b**), C/K (**c**), N/P (**d**), N/K (**e**), K/P (**f**), and Ca/Mg (**g**) of various components of the main herb plants in karst forests under different microhabitats. Different capital letters indicate significant differences among different components of plants in the same microhabitat (*p* < 0.05). The absence of any letter indicates that there is no significant difference between different plant components or different microhabitats (*p* > 0.05).

As shown in Figure 4, the stoichiometric ratios of the dominant herbs in karst forests’ leaves and roots did not show significant differences across the different microhabitats (*p* > 0.05). The stoichiometric ratios of plant components under the same microhabitat conditions were further analyzed. The results showed that the C/K of roots was significantly higher than that of leaves in all microhabitat types (*p* < 0.05). In addition, the C/P of roots was also significantly higher than that of leaves (*p* < 0.05) in the stone surface microhabitat, but the difference was not significant in the stone gully and soil surface microhabitat (*p* > 0.05). In contrast, C/N, N/P, N/K, K/P, and Ca/Mg showed relatively uniform distribution characteristics between plant leaves and roots, and the differences were not significant (*p* > 0.05).

### 2.3. Correlation Analysis Between Nutrient Contents and Stoichiometric Characteristics of Different Components of Main Shrubs and Herbs in Karst Forests

**Figure 5:**
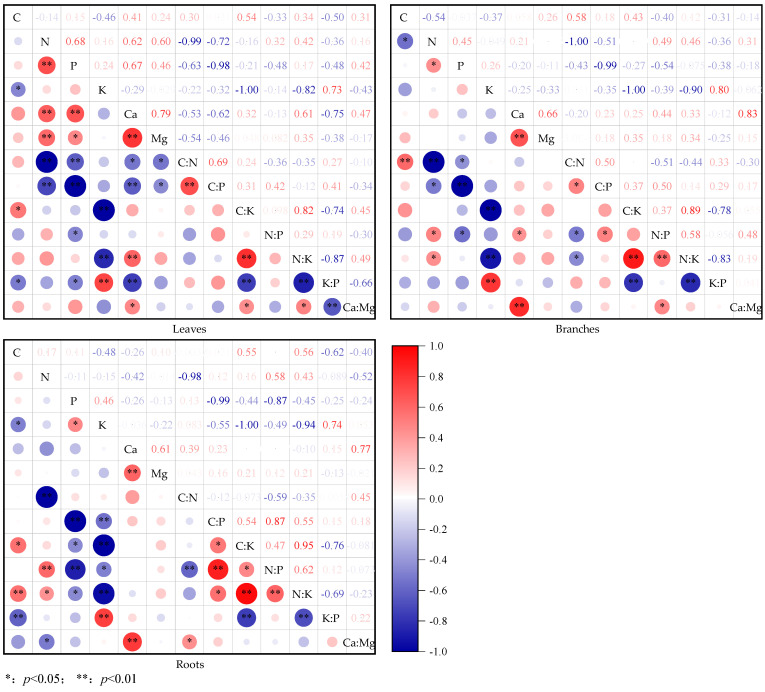
Correlation between the contents of C, N, P, K, Ca, and Mg in different components of the main shrub plants in karst forests and their stoichiometric ratios.

As shown in Figure 5, for the stoichiometric characteristics of shrub leaves, the positive correlation groups were as follows: C content was significantly (*p* < 0.05) positively correlated with C/K (r = 0.54); there were significant (*p* < 0.05) or extremely significant (*p* < 0.01) positive correlations between N content and P (r = 0.68), Ca (r = 0.62), and Mg (r = 0.60) contents, as well as between P content and Ca (r = 0.67) and Mg (r = 0.46) contents; K content was extremely significantly (*p* < 0.01) positively correlated with K/P (r = 0.73); Ca content was significantly (*p* < 0.05) or extremely significantly (*p* < 0.01) positively correlated with Mg (r = 0.79), N/K (r = 0.61), and Ca/Mg (r = 0.47). The correlations between stoichiometric ratios were as follows: C/N and C/P (r = 0.69) and C/K and N/K (r = 0.82) were extremely significantly (*p* < 0.01) positively correlated; Ca/Mg was significantly (*p* < 0.05) positively correlated with C/K (r = 0.45) and N/K (r = 0.49). The negative correlation group included the following: C content was significantly (*p* < 0.05) negatively correlated with K (r = 0.46) and K/P (r = 050); N content was extremely significant (*p* < 0.01) negatively correlated with C/N (r = 0.99) and C/P (r = 0.72); P content was significantly (*p* < 0.05) or extremely significantly (*p* < 0.01) negatively correlated with C/N (r = 0.63), C/P (r = 0.98), N/P (r = 0.48), and K/P (r = 0.49); K content was extremely significantly (*p* < 0.01) negatively correlated with C/K (r = 0.99) and N/K (r = 0.82) (*p* < 0.01). There were significant (*p* < 0.05) or extremely significant (*p* < 0.01) negative correlations between Ca content and C/N (r = 0.54) and C/P (r = 0.62), as well as between Mg content and C/N (r = 0.54) and C/P (r = 0.46). In addition, K/P was extremely significantly (*p* < 0.01) negatively correlated with C/K (r = 0.75), N/K (r = 0.88), and Ca/Mg (r = 0.66).

For the stoichiometric characteristics of shrub branches, the positive correlation groups include the following: C content was extremely significantly (*p* < 0.01) positively correlated with C/N (r = 0.58); N content was significantly (*p* < 0.05) positively correlated with P (r = 0.45), N/P (r = 0.49), and N/K (r = 0.46); K content was extremely significantly (*p* < 0.01) positively correlated with K/P (r = 0.80); Ca content was significantly (*p* < 0.05) or extremely significantly (*p* < 0.01) positively correlated with Mg (r = 0.66), N/P (r = 0.44), and Ca/Mg (r = 0.83). The correlations between stoichiometric ratios were as follows: C/P and C/N (r = 0.50), N/P (r = 0.50), and N/K and C/K (r = 0.89), and N/P (r = 0.59) showed significant (*p* < 0.05) or extremely significant (*p* < 0.01) positive correlation. The negative correlation group included the following: C content was significantly (*p* < 0.05) negatively correlated with N content (r = 0.54); there were significant (*p* < 0.05) or extremely significant (*p* < 0.01) negative correlations between N content and C/N (r = 0.99), C/P (r = 0.51), and P content and C/N (r = 0.944), C/P (r = 0.99); K content was extremely significantly (*p* < 0.01) negatively correlated with C/K (r = 0.99) and N/K (r = 0.90). In addition, C/N was significantly (*p* < 0.05) negatively correlated with N/P (r = 0.51) and N/K (r = 0.44); and K/P was extremely significantly (*p* < 0.01) negatively correlated with C/K (r = 0.78) and N/K (r = 0.83).

For stoichiometric characteristics of shrub roots, the positive correlation groups include the following: C content and C/K (r = 0.55), N/K (r = 0.56), N content and N/P (r = 0.58), and N/K (r = 0.43) showed significant (*p* < 0.05) or extremely significant (*p* < 0.01) positive correlations; there were significant (*p* < 0.05) or extremely significant (*p* < 0.01) positive correlations between P content and K content (r = 0.47), as well as between K content and K/P (r = 0.74); Ca content was extremely significantly (*p* < 0.01) positively correlated with Mg (r = 0.61) and Ca/Mg (r = 0.77). Correlations between stoichiometric ratios: C/P was significantly (*p* < 0.05) or extremely significantly (*p* < 0.01) positively correlated with C/K (r = 0.54), N/P (r = 0.87), and N/K (r = 0.55); C/K was significantly (*p* < 0.05) or extremely significantly (*p* < 0.01) positively correlated with N/P (r = 0.47) and N/K (r = 0.95); and N/P was extremely significantly (*p* < 0.01) positively correlated with N/K (r = 0.62). The negative correlation group included the following: C content was significantly (*p* < 0.05) or extremely significantly (*p* < 0.01) negatively correlated with K (r = 0.48) and K/P (r = 0.62); N content was significantly (*p* < 0.05) or extremely significantly (*p* < 0.01) negatively correlated with C/N (r = 0.99) and Ca/Mg (r = 0.52); and there were significant (*p* < 0.05) or extremely significant (*p* < 0.01) negative correlations between P content and C/P (r = 0.99), C/K (r = 0.44), N/P (r = 0.87), and N/K (r = 0.45), as well as between K content and C/P (r = 0.55), C/K (r = 0.99), N/P (r = 0.49), and N/K (r = 0.94). In addition, C/N and N/P (r = 0.59) and K/P and C/K (r = 0.76), N/K (r = 0.69) were extremely significantly (*p* < 0.01) negatively correlated.

**Figure 6:**
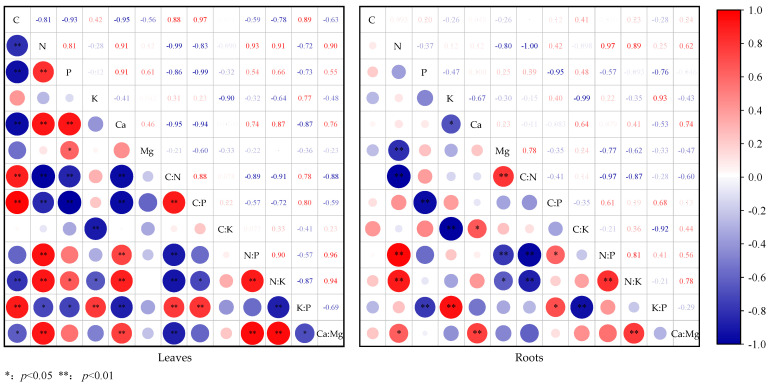
Correlation between the contents of C, N, P, K, Ca, and Mg in different components of the main herb plants in karst forests and their stoichiometric ratios.

For the stoichiometric characteristics of herbaceous leaves, there were obvious positive and negative correlation groups (Figure 6). The positive correlation groups were as follows: C content was extremely significantly (*p* < 0.01) positively correlated with C/N (r = 0.88), C/P (r = 0.97), and K/P (r = 0.89); N content was extremely significantly (*p* < 0.01) positively correlated with P (r = 0.81), Ca (r = 0.91), N/P (r = 0.93), N/K (r = 0.91), and Ca/Mg (r = 0.91); P content was significantly (*p* < 0.05) or extremely significantly (*p* < 0.01) positively correlated with Ca (r = 0.91), Mg (r = 0.61), and N/K (r = 0.66); K content was extremely significantly (*p* < 0.01) positively correlated with K/P (r = 0.77); and Ca content was extremely significantly (*p* < 0.01) positively correlated with N/P (r = 0.74), N/K (r = 0.87), and Ca/Mg (r = 0.76). The correlations between stoichiometric ratios were as follows: C/N was extremely significantly (*p* < 0.01) positively correlated with C/P (r = 0.88) and K/P (r = 0.78); C/P and K/P (r = 0.80) and N/K and Ca/Mg (r = 0.95) were extremely significantly (*p* < 0.01) positively correlated; N/P was extremely significantly (*p* < 0.01) positively correlated with N/K (r = 0.90) and Ca/Mg (r = 0.96). Negative correlation groups include the following: C content and N (r = 0.81), P (r = 0.93), Ca (r = 0.95), N/K (r = 0.78), and Ca/Mg (r = 0.63) were significantly (*p* < 0.05) or extremely significantly (*p* < 0.01) negative correlation; N content and C/N (r = 0.99), C/P (r = 0.83), and K/P (r = 0.72), P content and C/N (r = 0.86), C/P (r = 0.99), and K/P (r = 0.73), and Ca content and C/N (r = 0.95), C/P (r = 0.94), and K/P (r = 0.87) were all significantly (*p* < 0.05) or extremely significantly (*p* < 0.01) negative correlation; and K content was significantly (*p* < 0.05) or extremely significantly (*p* < 0.01) negatively correlated with C/K (r = 0.90) and N/K (r = 0.64). In addition, between stoichiometric ratios, the following correlations were apparent: C/N and N/P (r = 0.89), N/K (r = 0.91), and Ca/Mg (r = 0.88) were extremely significantly (*p* < 0.01) negative correlation; and N/K and K/P (r = 0.87) and K/P and Ca/Mg (r = 0.69) were significantly (*p* < 0.05) or extremely significantly (*p* < 0.01) negatively correlated.

For stoichiometric characteristics of herbaceous roots, the positive correlations among groups were as follows: N content was significantly (*p* < 0.05) or extremely significantly (*p* < 0.01) positively correlated with N/P (r = 0.97), N/K (r = 0.89), and Ca/Mg (r = 0.62); K content and K/P (r = 0.93) and Mg content and C/N (r = 0.78) were extremely significantly (*p* < 0.01) positively correlated; and Ca content was significantly (*p* < 0.05) or extremely significantly (*p* < 0.01) positively correlated with C/K (r = 0.64) and Ca/Mg (r = 0.74). The correlations between stoichiometric ratios were as follows: C/P was significantly (*p* < 0.05) positively correlated with N/P (r = 0.61) and K/P (r = 0.68); and N/K was extremely significantly (*p* < 0.01) positively correlated with N/P (r = 0.81) and Ca/Mg (r = 0.78). The negative correlation group included the following: N content was extremely significantly (*p* < 0.01) negatively correlated with Mg (r = 0.80) and C/N (r = 0.99); P content was extremely significantly (*p* < 0.01) negatively correlated with C/P (r = 0.95) and K/P (r = 0.76); K content was significantly (*p* < 0.05) or extremely significantly (*p* < 0.01) negatively correlated with Ca (r = 0.67) and C/K (r = 0.99); and Mg content was significantly (*p* < 0.05) or extremely significantly (*p* < 0.01) negatively correlated with N/P (r = 0.77) and N/K (r = 0.62). In addition, among stoichiometric ratios, C/N was extremely significantly (*p* < 0.01) negatively correlated with N/P (r = 0.97) and N/K (r = 0.87) (*p* < 0.01); and C/K was extremely significantly (*p* < 0.01) negatively correlated with K/P (r = 0.92).

### 2.4. Structural and Functional Traits of Leaves of Main Shrubs and Herbs in Karst Forests

According to the data of Table 1, the *SLA* and leaf water content (*LWC*) of shrubs in the soil surface microhabitat (180.03 ± 58.78 cm^2^·g^−1^ and 0.6317 ± 0.0740 g·g^−1^) were higher than those in the stone gully (166.70 ± 66.90 cm^2^·g^−1^ and 0.5931 ± 0.1180 g·g^−1^) and stone surface (177.11 ± 65.66 cm^2^·g^−1^ and 0.6119 ± 0.0945 g·g^−1^) microhabitats. The leaf dry matter content (*LDMC*) and *LTD* were the lowest in the soil surface microhabitat (0.3343 ± 0.0928 g·g^−1^ and 0.0451 ± 0.0155 g·cm^−3^). However, the variation trend in each herbaceous plant trait was the opposite to that of shrubs; the *SLA* and *LWC* of herbaceous plants were higher in the stone surface (294.70 ± 179.94 cm^2^·g^−1^ and 0.7444 ± 0.1557 g·g^−1^) and stone gully (222.54 ± 131.92 cm^2^·g^−1^ and 0.7533 ± 0.0952 g·g^−1^) microhabitat than in the soil surface (197.40 ± 99.95 cm^2^·g^−1^ and 0.7170 ± 0.1046 g·g^−1^) microhabitat. The *LDMC* and *LTD* were relatively low in the stone surface microhabitat (0.1985 ± 0.0783 g·g^−1^ and 0.0350 ± 0.0224 g·cm^−3^). However, these differences did not reach significant levels among different microhabitats (*p* > 0.05). From the perspective of plant forms, in all microhabitat types, the *LDMC* of shrubs (0.3343 ± 0.0928 g·g^−1^—0.3775 ± 0.1159 g·g^−1^) was significantly (*p* < 0.05) higher than that of herbs (0.1985 ± 0.0783 g·g^−1^—0.2206 ± 0.0458 g·g^−1^), with a difference range of 52% to 76%. However, the *SLA* (197.40 ± 99.95 cm^2^·g^−1^—294.70 ± 179.94 cm^2^·g^−1^) and *LWC* (0.7170 ± 0.1046 g·g^−1^—0.7533 ± 0.0952 g·g^−1^) of herbs were generally higher than those of shrubs (*SLA*: 166.70 ± 66.90 cm^2^·g^−1^—180.03 ± 58.78 cm^2^·g^−1^; *LWC*: 0.5931 ± 0.1180 g·g^−1^—0.6317 ± 0.0740 g·g^−1^), but the differences were not significant (*p* > 0.05).

It can be seen from Table 2 that, under different microhabitat conditions, the net photosynthetic rate (*P_n_*), stomatal conductance (*G_s_*), intercellular CO_2_ concentration (*C_i_*), and transpiration rate (*T_r_*) of shrubs generally showed that the stone surface microhabitat (4.8628 ± 1.3556 μmol·m^−2^·s^−1^, 0.1149 ± 0.1242 mol·m^−2^·s^−1^, 276.77 ± 89.60 μmol·mol^−1^, and 0.9143 ± 0.4548 mmol·m^−2^·s^−1^) was higher than the soil surface (4.7126 ± 1.2368 μmol·m^−2^·s^−1^, 0.0940 ± 0.0720 mol·m^−2^·s^−1^, 286.34 ± 74.36 μmol·mol^−1^, and 0.8294 ± 0.2187 mmol·m^−2^·s^−1^) and the stone gully (4.6421 ± 1.0079 μmol·m^−2^·s^−1^, 0.0698 ± 0.0464 mol·m^−2^·s^−1^, 265.13 ± 60.45 μmol·mol^−1^, and 0.7573 ± 0.1932 mmol·m^−2^·s^−1^) microhabitats; the *P_n_*, *G_s_*, *C_i_*, and *T_r_* of herbaceous plants were consistent with those of shrubs, and they were also the highest in the stone surface microhabitat (3.0908 ± 1.4858 μmol·m^−2^·s^−1^, 0.1088 ± 0.0986 mol·m^−2^·s^−1^, 295.84 ± 101.43 μmol·mol^−1^, and 0.8104 ± 0.4147 mmol·m^−2^·s^−1^), being lower in the soil surface (2.3266 ± 1.8120 μmol·m^−2^·s^−1^, 0.0596 ± 0.0792 mol·m^−2^·s^−1^, 273.94 ± 69.17 μmol·mol^−1^, and 0.5603 ± 0.3270 mmol·m^−2^·s^−1^) and stone gully (2.5062 ± 1.1678 μmol·m^−2^·s^−1^, 0.0599 ± 0.0691 mol·m^−2^·s^−1^, 258.14 ± 91.04 μmol·mol^−1^, and 0.5350 ± 0.3006 mmol·m^−2^·s^−1^) microhabitats; however, these differences did not reach significant levels among the different microhabitats (*p* > 0.05). From the perspective of the plant life form, the *P_n_* of shrubs in the stone gully (4.6421 ± 1.0079 μmol·m^−2^·s^−1^), stone surface (4.8628 ± 1.3556 μmol·m^−2^·s^−1^), and soil surface (4.7126 ± 1.2368 μmol·m^−2^·s^−1^) microhabitats were higher than that of herbs (2.5062 ± 1.1678 μmol·m^−2^·s^−1^, 3.0908 ± 1.4858 μmol·m^−2^·s^−1^, and 2.3266 ± 1.8120 μmol·m^−2^·s^−1^); the differences were 85%, 57%, and 103%, respectively, and the difference in the stone gully and soil surface microhabitats reached a significant level (*p* < 0.05). The *G_s_* and *T_r_* of shrubs were higher than those of herbs across all microhabitat types. Additionally, the *C_i_* of shrubs was higher than that of herbs in the stone gully and soil surface microhabitats but slightly lower in the stone surface microhabitat. However, the differences between the two plant forms were not significant (*p* > 0.05).

### 2.5. Effects of Environmental Factors on Stoichiometric Characteristics of Different Components of the Main Shrubs and Herbs in Karst Forests

#### 2.5.1. Redundancy Analysis

The RDA results showed that there were significant differences (*p* < 0.05) between the first ordination axes of different components of shrubs and herbs and other ordination axes, indicating that the ordination results were reliable (Tables S1 and S2). Among them, the total explanation rates of the effects of environmental factors on the stoichiometric characteristics of shrub leaves, branches, and roots were 70.32%, 57.30%, and 60.47%, respectively, and the first ordination axis explained 40.97%, 32.60%, and 37.45%, respectively; the total explanation rates for herb leaves and roots were 85.97% and 74.29%, respectively, and the first ordination axis explained 66.42% and 42.01%, respectively. In summary, the first two ordination axes of RDA can effectively characterize the correlation between the stoichiometric characteristics of plant components and environmental factors, among which the first ordination axis contributes the most.

**Figure 7:**
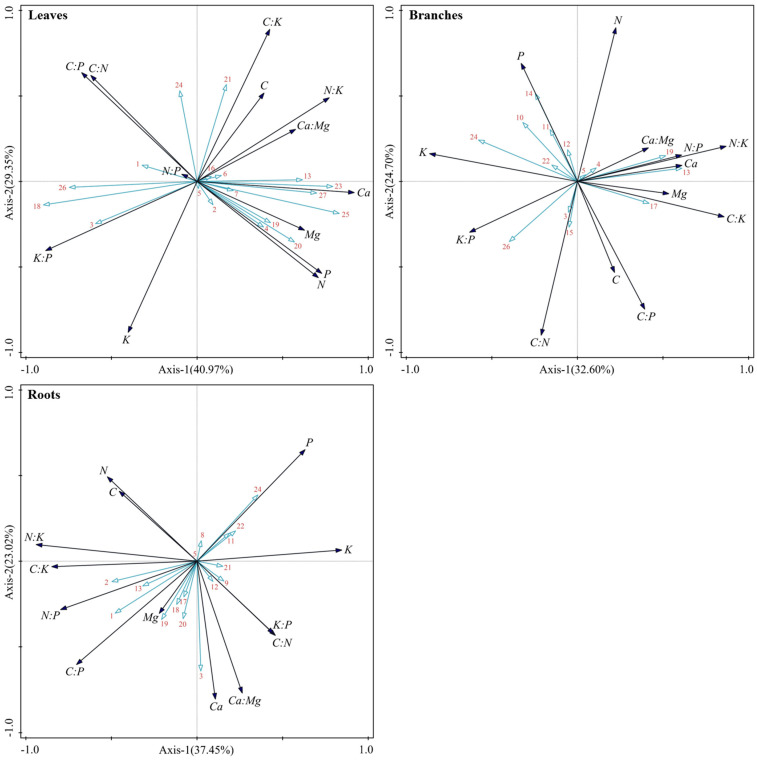
RDA ordination diagram of stoichiometric characteristics and environmental factors concerning different components of the main shrub plants in karst forests. The numbers 1–5 represent the topographic factors of elevation, slope, aspect, slope position, and microhabitat, respectively. The numbers 6–17 represent the total soil N, hydrolytic N, total P, available P, total K, available K, total Ca, exchangeable Ca, total Mg, exchangeable Mg, organic carbon, and pH, respectively. The numbers 18–28 represent the plant species, ground diameter, plant height, *P_n_*, *G_s_*, *C_i_*, *T_r_*, *SLA*, *LDMC*, *LWC*, and *LTD*, respectively.

**Figure 8:**
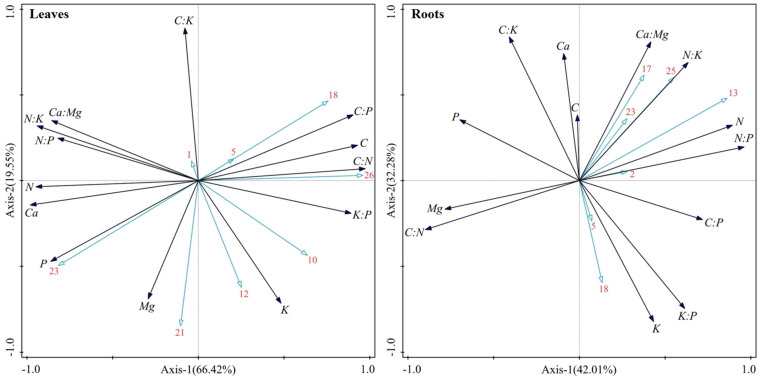
RDA ordination diagram of stoichiometric characteristics and environmental factors of different components of the main herb plants in karst forests. The numbers 1, 2, and 5 represent the topographic factors elevation, slope, and microhabitat, respectively. The numbers 10, 12, 13, and 17 represent the total K, total Ca, exchangeable Ca, and pH, respectively. The numbers 18, 21, 23, 25, and 26 represent the plant species, *C_i_*, *SLA*, *LWC*, and *LTD*, respectively.

The RDA ordination diagram of the stoichiometric characteristics and environmental factors concerning shrubs and herbs was further analyzed. The results showed that the species attributes of shrubs were positively correlated with leaf K content and C/N, C/P, and K/P and negatively correlated with N, P, Ca, and Mg content and C/K, N/K, and Ca/Mg, but there was little correlation with C content and N/P. *T_r_* was positively correlated with P content in branches and negatively correlated with Ca content, C/P, N/P, and Ca/Mg, but there was little effect on N content and C/N. The elevation gradient was positively correlated with the N content and C/P and N/P of the root system, negatively correlated with the P content and C/N, and less correlated with the Ca content and Ca/Mg (Figure 7). *LTD* was positively correlated with leaf C content and C/N, C/P, and K/P and negatively correlated with N, P, and Ca content and N/P, N/K, and Ca/Mg, but there was little effect on C/K. Soil-exchangeable Ca was positively correlated with root N content and N/P, N/K, and Ca/Mg and negatively correlated with Mg content and C/N, but there was little correlation with K content and C/K (Figure 8).

The individual benefit ranking analysis based on the Monte Carlo permutation test (999 permutations) (Table S3) showed that the stoichiometric characteristics of karst forest plants showed significant component-specific regulation patterns, and the environmental driving factors of different components showed multi-dimensional synergistic effects. The stoichiometric characteristics of shrub leaves were mainly regulated by plant species (explained 34.3%, *p* < 0.01) and formed a synergistic driving effect with *P_n_* (13.7%, *p* < 0.01) and slope position (10.7%, *p* < 0.01). The branches were mainly dominated by *T_r_* (19.4%, *p* < 0.01) and soil-exchangeable Ca (16.6%, *p* < 0.01). Root characteristics showed a multi-factor synergistic regulation effect: altitude gradient (18.2%, *p* < 0.01), soil-exchangeable Ca (15.3%, *p* < 0.01), and total Ca (14.0%, *p* < 0.01) were the core drivers and were significantly affected by slope aspect (12.9%, *p* < 0.05). The stoichiometric characteristics of herb leaves were absolutely dominated by *LTD* (61.5%, *p* < 0.01) and were synergistically affected by *SLA* (15.6%, *p* < 0.05) and plant species (11.6%, *p* < 0.05). Root characteristics were mainly controlled by soil-exchangeable Ca (38.7%, *p* < 0.01) and had a significant synergistic effect with plant species (17.6%, *p* < 0.05) and *SLA* (17.3%, *p* < 0.05).

#### 2.5.2. Variance Partitioning Analysis

**Figure 9:**
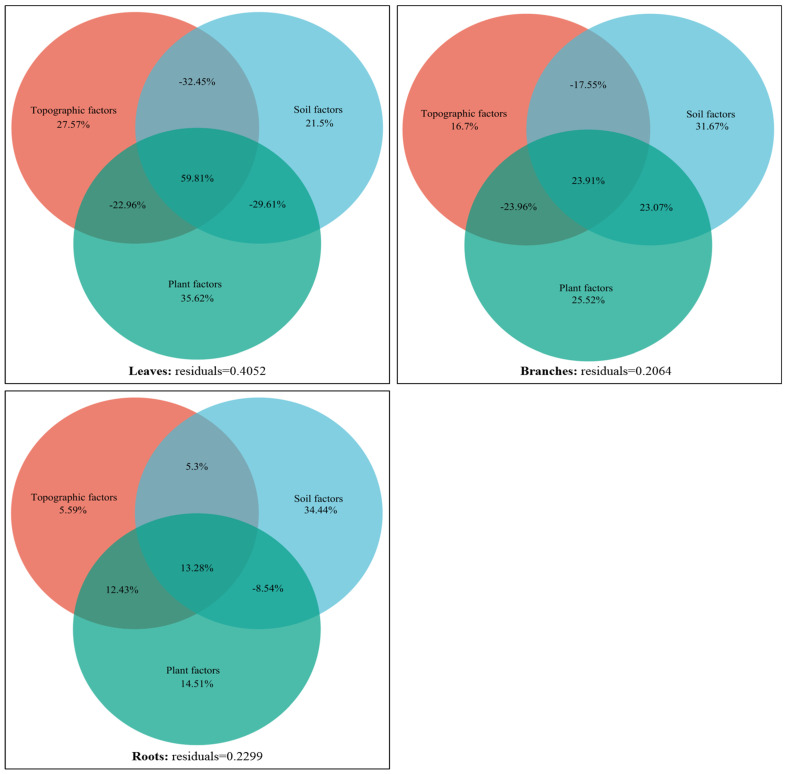
VPA results of the stoichiometric characteristics of different components of the main shrub plants in karst forests under the influence of environmental factors.

The environmental driving mechanism of the variation in the stoichiometric characteristics of the dominant plants in karst forests was further revealed by VPA. The results showed that there were significant differences in the environmental driving patterns of shrubs and herbs. The total explanation of the effects of environmental factors on the variation in shrub leaves, branches, and roots was 59.48%, 79.36%, and 77.01%, respectively (Figure 9). In terms of individual effects, plant characteristics contributed the most to leaf variation (35.62%), while soil traits were the main independent driving force for branch (31.67%) and root (34.44%) variation. In the two-factor interaction, the synergistic explanation of plant × soil on branch variation was the highest (23.07%), and the interaction of topography × plant was also important for root variation (12.43%). The negative values of some two-factor interactions (such as the influence of topography × plant on leaves and branches) were mainly due to the instability of model estimation caused by the multicollinearity among environmental factors, and its practical ecological significance could be ignored. In addition, the synergistic effect of the three factors (topography × soil × plant) explained 59.81%, 23.91%, and 13.28% of the variation in shrub leaves, branches, and roots, respectively.

**Figure 10:**
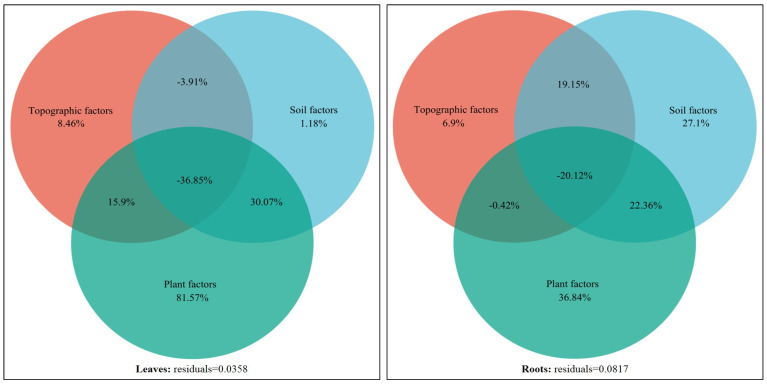
VPA results of the stoichiometric characteristics of different components of the main herb plants in karst forests under the influence of environmental factors.

In contrast, the overall explanation of the effects environmental factors on the variation in herbaceous leaves and roots was higher, reaching 96.42% and 91.83%, respectively (Figure 10). Among them, the individual effects of plant characteristics were clearly dominant, independently explaining 81.57% and 36.84% of the variation in leaves and roots, respectively. At the same time, the contribution of plant × soil two-factor interaction to leaf (30.07%) and root (22.36%) variation was also very prominent. The synergistic effect of the three factors is extremely low in the interpretation of each component of the herb.

## 3. Discussion

### 3.1. Distribution Characteristics of Nutrient Contents and Stoichiometric Ratios in Different Components of Shrubs and Herbs in Karst Forests at Microhabitat Scale

Plant growth and development are closely related to environmental factors. The dynamic regulation of resource allocation pattern and ecological stoichiometric characteristics of each component is an important physiological and ecological strategy for plants to cope with environmental stress [17].

This study found that the contents of C, N, P, K, Ca, and Mg (Figure 1 and Figure 2) and their stoichiometric ratios (Figure 3 and Figure 4) in the components of the dominant shrubs and herbs in karst forests were not significantly different across different microhabitats (*p* > 0.05). This phenomenon can be preliminarily discussed from the following perspectives: (1) Although the surface heterogeneity of the karst area is strong, its soil is developed from limestone soil formed by the weathering of carbonate rocks. The relative homogeneity of this soil formation process (Table S5) may reduce the difference in soil chemical properties between microhabitats to a certain extent, thus weakening the variation in plant stoichiometric characteristics. (2) Plants may transform the heterogeneity of the external environment into internal metabolism heterogeneity through stoichiometric homeostasis regulation (such as enhancing the nutrient reabsorption rate of leaves), component function differentiation (such as higher C investment in roots), and root configuration optimization [18] (reducing fine root secondary branches and extending fine root connection length), thus weakening the influence of microhabitat differentiation on plant stoichiometric characteristics, but the specific mechanism needs to be further verified.

Further analysis of the distribution of elements in each component showed that the contents of N, P, K, and Mg in shrub leaves were significantly higher than those in branches and roots (*p* < 0.05) (Figure 1), while C/N, C/P, and C/K showed the opposite trend (Figure 3). This finding is basically consistent with existing research conclusions [19]. This specific distribution pattern is mainly due to the functional differentiation of plant components and the different resource allocation strategies. As the core organ of photosynthesis and material production, the high metabolic demand of leaves determines the priority allocation of key elements such as N, P, K, and Mg, among which N and P elements are directly involved in protein synthesis, chloroplast construction, and genetic material formation [20]. K is essential for leaf water balance and metabolic homeostasis under karst drought stress through the regulation of stomatal opening and closing, maintaining cell osmotic pressure balance and activating enzyme activity [21]. As the core component of chlorophyll, Mg is directly involved in the light energy capture process [22]. In contrast, branches and roots are structural organs, and the structural carbon components such as cellulose and lignin in xylem and phloem account for a relatively high proportion. While undertaking mechanical support and nutrient storage functions, they form a C content level similar to that of leaves (Figure 1a). The balance of this carbon skeleton investment is in stark contrast to the unbalanced distribution of N, P, K, and other nutrient elements. Plants preferentially allocate N, P, K, and other elements to metabolically active organs (such as leaves) and actively reduce the nutrient content of branches and roots to construct higher C/N, C/P, and C/K, which not only ensures the stable accumulation of structural carbon but also avoids the redundant retention of nutrients in non-metabolic organs.

Furthermore, the K content of herb leaves was significantly higher than that of roots in different microhabitats (*p* < 0.05), which was closely related to the rapid growth strategy of herbs. K^+^ drives the elongation growth of the aboveground part by maintaining cell turgor pressure [21], and it is essential for the competitive strategy of herbaceous plants. However, there was no significant difference in N/P, N/K, K/P, and Ca/Mg among different components of shrubs and herbs (*p* > 0.05), which revealed that plants followed a strict homeostasis mechanism in the process of nutrient regulation [23]. Through root selective absorption and in vivo transport regulation, plants dynamically adjust the absorption ratio and distribution pattern of each element to ensure that the nutrient supply of different components matches their physiological function needs so as to avoid excessive accumulation or deficiency of a single element, forming a stoichiometric regulation network adapted to karst special habitats.

### 3.2. Coupling Relationship Between Nutrient Content and Stoichiometric Characteristics of Different Components of Shrubs and Herbs in Karst Forests at Microhabitat Scale

The absorption and utilization of mineral elements by plants often follow a certain proportional relationship. The interaction between these elements maintains the ion balance and metabolic homeostasis in plants through antagonism and synergistic effects [24]. Elements with similar physicochemical properties may form competitive inhibition in absorption sites or metabolic pathways, while elements with complementary functions may produce synergistic effects by sharing transporters or participating in the same biochemical reaction. This dynamic element interaction mechanism helps to improve the nutrient use efficiency of plants and is an optimal resource allocation strategy formed by plants during long-term evolution [25].

The results of this study showed that there was a significant (*p* < 0.05) or extremely significant (*p* < 0.01) positive correlation between N, P, Ca, and Mg elements in shrub leaves, which may be related to the investment of plants in photosynthetic organs and the functional synergy in the construction of cell structure [26], which is also the key physiological basis for shrubs in adapting to the heterogeneous karst environment. They were significantly (*p* < 0.05) or extremely significantly (*p* < 0.01) negatively correlated with C/N and C/P, which may be related to the reabsorption of N and P during leaf senescence [27]; in addition, structural carbon is difficult to decompose, resulting in the relative enrichment of N and P in leaves, thereby reducing C/N and C/P. Leaf C was significantly (*p* < 0.05) positively correlated with C/K, revealing that in karst habitats where K^+^ is easy to leach [28], plants may partially compensate for the lack of K^+^ in osmotic regulation and other functions by increasing structural carbon accumulation.

Despite C being significantly (*p* < 0.05) negatively correlated with N and P in plant leaves [29], there was no significant correlation between C and N and P in shrub leaves in this study (*p* > 0.05). This phenomenon may be related to its genetic characteristics and nutrient utilization strategies. Higher C/N and C/P are helpful in improving the utilization efficiency of N and P [30] and alleviating the dilution effect of C accumulation on nutrients. At the same time, the deep penetration of shrub roots can directly obtain the N and P elements released by bedrock weathering [31], which breaks through the limitation of nutrient supply in the topsoil and realizes the decoupling of nutrient absorption and C accumulation in time and space. In contrast, C in herbaceous leaves was extremely significantly (*p* < 0.01) negatively correlated with N and P; N and P were also extremely significantly (*p* < 0.01) negatively correlated with C/N and C/P; while C was extremely significantly (*p* < 0.01) positively correlated with C/N and C/P, which was consistent with the “element dilution effect” under the rapid growth strategy of herbaceous plants; that is, the photosynthetic carbon fixation rate exceeded the N and P nutrient acquisition rate, resulting in a decrease in N and P content per unit biomass [29].

In the branches of shrubs, C and N showed a significant (*p* < 0.05) negative correlation, while C and C/N, as well as C/P and C/N, N/P, were significantly (*p* < 0.05) or extremely significantly (*p* < 0.01) positively correlated, reflecting that as a structural organ, branches mainly accumulate C, and at the same time, it also indicates the process of nutrient transfer and redistribution. Additionally, under nutrient-limited conditions, the C accumulation rate was much higher than the N and P acquisition rates, this asymmetric growth led to the synchronous increase in C/N and C/P; N/P increased synergistically due to the high N absorption efficiency. Ca was significantly (*p* < 0.05) or extremely significantly (*p* < 0.01) positively correlated with Mg, N/P, and Ca/Mg, which might be related to the maintenance of ionic homeostasis and the strengthening of cell wall function in the high-Ca and low-P karst environment [26,32]. The regulation of N/P also reflects that plants maintain metabolic balance by improving nitrogen use efficiency in this environment [33].

C in shrub roots was significantly (*p* < 0.05) or extremely significantly (*p* < 0.01) positively correlated with C/K and N/K and significantly (*p* < 0.05) or extremely significantly (*p* < 0.01) negatively correlated with K and K/P, which may reflect the distribution strategy of C under K limitation; that is, the root system preferentially sequesters structural carbon to enhance its ability to penetrate rock crevices to obtain deep nutrients [34]. At the same time, limited by the slow-release characteristics of K and ion competition [35,36], K^+^ was preferentially transported to aboveground photosynthetic organs, reducing root retention to achieve metabolic efficiency optimization, resulting in lower K content in roots (Figure 1d), which indirectly increased C/K and N/K, reflecting the “mechanical resistance priority–metabolic cost optimization” survival strategy of shrubs in barren habitats. N was significantly (*p* < 0.05) or extremely significantly (*p* < 0.01) positively correlated with N/P and N/K and significantly (*p* < 0.05) or extremely significantly (*p* < 0.01) negatively correlated with C/N and Ca/Mg. This study revealed the internal coupling relationship between N metabolism and P, K, Ca, Mg, and other elements: under the condition of P limitation, plants tend to improve N utilization efficiency to promote N accumulation and N/P synchronous rise. At the same time, mycorrhiza assisted N acquisition to reduce the cost of C metabolism, thereby reducing C/N. Mg deficiency limits chlorophyll synthesis [22] and indirectly weakens N assimilation capacity, resulting in a significant negative correlation between N and Ca/Mg. In addition, the significantly (*p* < 0.05) positive correlation between P and K may be due to their synergistic effect in energy metabolism [36].

In herbaceous roots, N was extremely significantly (*p* < 0.01) negatively correlated with Mg and C/N, and Mg was extremely significantly (*p* < 0.01) positively correlated with C/N, which may be related to the preferential distribution strategy of Mg to photosynthetic organs and its regulation via nitrogen metabolism [33]. However, K was significantly (*p* < 0.05) or extremely significantly (*p* < 0.01) negatively correlated with Ca and C/K, while Ca was significantly (*p* < 0.05) positively correlated with C/K, which revealed the antagonistic mechanism of Ca-K ions: under a high-Ca environment, Ca^2+^, as the second messenger in plants, inhibits the absorption of K^+^ by competing for binding sites on the plasma membrane [37]; the lack of K forces plants to maintain cell osmotic balance through the redistribution of C resources, which jointly drives the increase in C/K.

### 3.3. Factors Affecting the Stoichiometric Characteristics of Different Components of the Dominant Shrubs and Herbs in Karst Forests and Their Adaptation Mechanisms

At the microhabitat scale, dynamic changes in the element contents and stoichiometric ratios of the different components of the dominant shrubs and herbs in karst forests result from the interactions between biological genetic characteristics and abiotic environmental factors, which reflects the differences in the acquisition strategies of different plant forms with respect to heterogeneous habitat resources, and it is also a kind of adaptation mechanism in response to a complex and changeable environment.

This study shows that the stoichiometric characteristics of shrub leaves are mainly controlled by plant species (34.3%) (Figure 7 and Table S3), which is basically consistent with the research conclusions of Sardans et al. [38], Tian et al. [39], and Valicrosa et al. [40], highlighting the importance of species categories in shaping plant nutrient utilization strategies. In contrast, Zhang et al. [41], based on the study of large-scale transects in China, emphasized the dominant role of climatic factors (such as temperature and precipitation) in leaf element content. This difference is mainly due to the different research scales: species specificity is more pronounced in local microhabitats, while climate filtering dominates at the regional scale. The stoichiometric characteristics of herbaceous leaves were mainly regulated by *LTD* (61.5%) (Figure 8 and Table S3). The C content was significantly (*p* < 0.05) positively correlated with *LTD*, which may be related to the rapid growth strategy adopted by herbaceous plants. It optimizes leaf structure by thickening the cuticle and cell wall and maintains high photosynthetic C fixation efficiency while improving water use efficiency and stress resistance. In addition, *SLA* was significantly (*p* < 0.05) positively correlated with leaf N and P content, which further indicated that herbaceous plants expanded light capture area through thin leaf morphology (high *SLA*) to support their rapid growth strategy. The stoichiometric characteristics of shrub branches are dominated by *T_r_* and soil-exchangeable Ca. Shrubs maintain the transport of water and nutrients through transpiration [42] and are significantly (*p* < 0.05) positively correlated with P content, indicating that this drives the upward transport of P in xylem through transpiration tension. Soil-exchangeable Ca may enhance the mechanical strength of the branch cell wall via the cross-linking of calcium pectate [26], affecting secondary xylem development and mechanical support function. The stoichiometric characteristics of roots were significantly affected by soil-exchangeable Ca in shrubs and herbs, but the mechanism and degree were different across different plant forms. P in karst soil is easy to fix as insoluble Ca-P compounds, which reduces the effectiveness of P [43]. Plants alleviate P limitation by root exudates [35,44]. Herbs rely on shallow roots with high metabolic activity to activate surface soil P, so they are more affected by exchangeable Ca (38.7%). Shrubs obtain slow-release P in rock crevices through deep roots and are less dependent on exchangeable Ca (15.3%). In addition, exchangeable Ca can also affect the absorption of other nutrients by regulating soil cation balance [45].

The results of VPA further showed that (Figure 9 and Figure 10) shrubs and herbs had obvious strategic differentiation in terms of the “high-Ca–low-P heterogeneity” environmental driving mode in karst. As a perennial woody plant, shrubs rely on deep roots to directly obtain slow-release nutrients in rock crevices in order to meet the resource needs of long-term growth. Their nutrient supply is synergistically regulated by root architecture [18], topographic factors, and soil-exchangeable Ca, resulting in a synergistic effect of topography × soil × plant, which contributes 59.81% and 13.28% to the stoichiometric characteristics of leaves and roots, respectively. The branches were more regulated by soil traits (31.67%) and plant characteristics (25.52%) alone, and the balance between structural support and nutrient acquisition was achieved through the interaction of plant × soil (23.07%). This reflects that shrub plants in the karst area mainly deal with complex terrain-soil system through component functional differentiation. In contrast, herbaceous plants, due to their short life cycle and flexible reproductive strategies, rely mainly on phenotypic plasticity to respond quickly to environmental changes. They maximize light energy capture efficiency through high *SLA* and low *LTD*, and they rely on shallow roots with high metabolic activity to activate surface soil P [44] to support its rapid growth. The stoichiometric characteristics of leaves and roots were mainly dominated by plant characteristics (explained by 81.57% and 36.84%, respectively), and the dynamic balance of available nutrient uptake and carbon metabolism distribution was achieved through plant × soil interaction (leaves 30.07%, roots 22.36%). The lack of a synergistic effect of terrain × soil × plant further confirms that herbaceous plants are more inclined to simplify the environmental response network—a niche rapid turnover strategy.

This study also found that there were significant differences in the degree of interpretation of environmental factors on the stoichiometric characteristics of different plant forms. Among them, herbaceous leaves and roots were the highest (all >90%) (Figure 10), followed by shrubs (leaves, branches, and roots were 59.48%, 79.36%, and 77.01%, respectively) (Figure 9), and the lowest were tree components (33.69–64.25%) [16]. This may be due to the combined effects of life history strategies and niche differentiation: herbaceous plants have short growth cycles, high plasticity, and are more sensitive to environmental responses; tree species grow slowly and have high stability, and there is a significant lag effect on their response to environmental changes, resulting in the relatively weak impact of environmental factors. In addition, in ecosystems, herbaceous plants tend to be at a lower trophic level and face more intense resource competition, so they show higher sensitivity to changes in environmental factors. Due to their large size, tree species can obtain more light and space resources through competition, thus reducing their dependence on environmental factors. The aspects of the spatial variation in plant stoichiometric characteristics that are unexplained by biotic and abiotic factors may be caused by an internal regulatory mechanism formed by plants in the community to avoid interspecific competition [38] that reduces the overlap of resource utilization through the differentiation of stoichiometric characteristics.

## 4. Materials and Methods

### 4.1. Study Area

The study area is located in Maolan National Nature Reserve, Libo County, Qiannan Prefecture, Guizhou Province. It is located in the slope zone of the transition from the Yunnan–Guizhou Plateau to the Guangxi hilly plain. The geographical coordinates are 107°52′10″–108°05′40″ E, 25°09′20″–25°20′50″ N [46]. The terrain in the area is high in the northwest and low in the southeast. The highest altitude is 1078.6 m, the lowest is 430.0 m, and the average is between 550 and 850 m. The region features a typical subtropical monsoon humid climate, characterized by warm springs and autumns, mild winters, moderate summers, and ample precipitation. The annual average temperature is 15.3 °C, the average temperature in January is 5.2 °C, the average temperature in July is 23.5 °C, the annual precipitation is 1752.5 mm, the annual average relative humidity is 83.0%, the annual sunshine hours is 1272.8 h, the sunshine percentage is 29.0%, the frost-free period is 315 days, the annual total solar radiation is 63 289.80 kW/m^2^, and the accumulated temperature ≥ 10 °C is 4598.6 °C. The karst landform in the area is highly developed, and the exposed rate of bedrock is more than 80%. The predominant geomorphological types are peak cluster funnels and peak cluster depressions, formed primarily in limestone and dolomite [47]. The soil type is mainly lime soil formed by the weathering of carbonate rocks. It has the characteristics of a shallow soil layer, rare soil, and discontinuous distribution. It mainly occurs in the rock gap and has rich organic matter content. The vegetation in the study area is dominated by the primary evergreen deciduous broad-leaved mixed forest developed on the karst landform. As a non-zonal vegetation [47], the community structure is complete, and the differentiation between the tree layer, shrub layer, and herb layer is clear. The dominant tree species are *Cyclobalanopsis glauca* (Thunb.) Oerst., *Platycarya strobilacea* Siebold and Zucc., *Acer wangchii* Fang., and *Cornus wilsoniana* Wangerin, etc. The shrub plants mainly include *Nandina domestica* Thunb., *Brassaiopsis glomerulata* (Blume) Regel., *Miliusa sinensis* Finet and Gagnep., *Mahonia cardiophylla* T. S. Ying and Boufford, etc. Herb plants are mainly *Cyperus rotundus* L., *Strobilanthes maolanensis* Blume., *Pilea cavaleriei* H. Lév., and *Asplenium nidus* L., etc. [16]. The forest coverage rate is as high as 87.4%.

### 4.2. Study Methods

#### 4.2.1. Plot Setting

**Figure 11:**
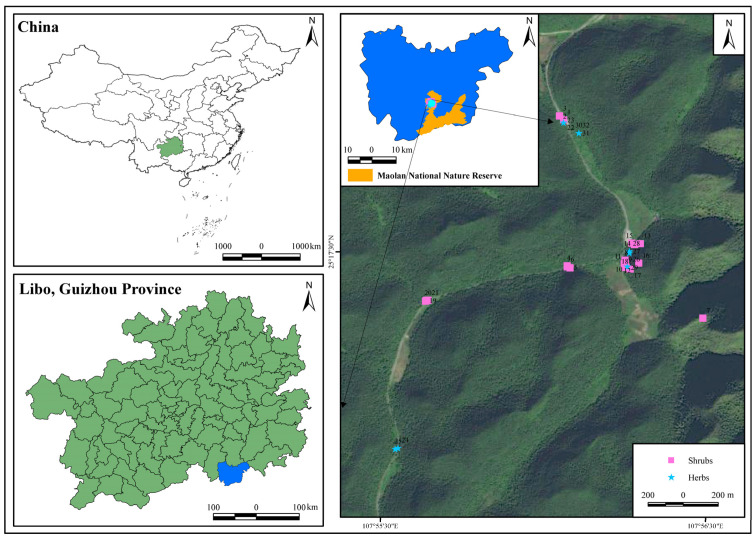
Diagram of plant sample locations. Numbers 1–32 represent each plant sample.

Based on the analysis of the importance of species in the fixed monitoring plots of the Guizhou Libo Karst Forest Ecosystem Observation and Research Station, this study selected 7 dominant shrubs and 3 dominant herbs as the research objects. According to the microhabitat type of plant roots, combined with the distribution characteristics of different slope positions, aspects, and degrees (categorized as follows: Flat slope ≤ 5, gentle slope 5–15°, moderate slope 15–25°, steep slope 25–35°, and very steep slope ≥ 35°), and on the premise of ensuring that each plant is represented by no fewer than 3 individuals, a total of 32 sampling points were established (Figure 11). At each sampling point, the latitude, longitude, and altitude of each sample plant were accurately determined by a hand-held GPS (Garmin Montana 680; positioning accuracy: 3–5 m), the slope and aspect were accurately measured by a compass (DQL-12; horizontal calibration has been performed in a non-magnetic interference environment before use), and the slope grade was divided via visual observation. The species name, plant height, ground diameter, and microhabitat characteristics of the shrub plants were recorded in detail. For herbs, we focused on recording their species names and their corresponding microhabitat types (Table S4).

#### 4.2.2. Microhabitat Division

This study is mainly based on the classification method of karst forests microhabitat types proposed by Zhu et al. [8]. According to the causes and external morphological characteristics of the microhabitat, combined with its typicality, particularity, and field operability, we divide it into three types: stone gully, stone surface, and soil surface. The characteristics of the different microhabitat types and soil chemical properties are detailed in Table S5.

#### 4.2.3. Sampling

- Leaves: Collected from August to October 2020. Using branch scissors (plant height ≤ 2 m) or high branch scissors (plant height > 2 m), the branches with good growth in the east, south, west, and north directions and the upper, middle, and lower parts of each shrub plant were cut, respectively, and the leaves with complete extension, no disease, and no petiole on the branches were picked. The picked leaves were fully and evenly mixed, and 10–20 samples were retained by the quartering method. The samples were packaged with a self-sealing bag number and placed in a portable refrigerator for use. Each plant was a sample, and all traits were measured independently without sample mixing. The same is true for branch, root, and soil samples.
- Branches: The trunks, lateral branches, and sprouting branches of each shrub plant were intercepted by branch scissors or high branch scissors, and the excess leaves and terminal parts were removed. After being fully mixed, samples of no less than 100 g were retained by the quartering method, numbered, and packaged with self-sealing bags and placed in a portable refrigerator for later use.
- Roots: Tall shrubs were excavated by an in situ layered excavation method. After removing the litter layer on the surface, a stainless steel root shovel was used for layer by layer excavation along the root direction by a progressive stripping method, and the depth was excavated to 30–50 cm. The complete root segment with a length of 5–8 cm was cut directly by a branch shear. For dwarf shrubs and herbs, we used the whole root excavation method to collect root samples. Samples were packaged with a self-sealing bag number and placed in a portable refrigerator for later use.
- Soil: The surface litter and gravel around the roots of each plant were removed, and the surface soil at a depth of 0–20 cm was collected with a soil sampler. The soil around the roots of the same plant was fully mixed, and impurities such as impurities and roots were removed. A sample of no less than 500 g was retained by the quartering method, and the soil bag was numbered and packaged and placed in a portable refrigerator for later use.

#### 4.2.4. Determination of Plant Structural and Functional Traits

- Plant structural traits:
  (1) Leaf fresh weight (*LFW*, g): Five representative leaf samples were selected for each individual plant, and they were weighed using an electronic balance with an accuracy of 1/10,000.
  (2) Leaf thickness (*LT*, mm): Three points were evenly selected along the main vein of the leaf using a digital vernier caliper and measured separately.
  (3) Leaf turgid weight (*LTW*, g): The leaves were soaked in clean water for 24 h in a dark environment to fully absorb water and saturate. After the water on the surface of the leaves was quickly removed with absorbent paper, they were weighed using an electronic balance.
  (4) Leaf area (*LA*, cm^2^): Leaf area was measured using a portable leaf area meter (LI–3100, LI–Cor Inc., Lincoln, NE, USA) [48].
  (5) Leaf dry weight (*LDW*, g): The leaves were dried in an oven at 65 °C for 72 h until a constant weight was reached, and they were weighed using an electronic balance.
  (6) *SLA* (cm^2^·g^−1^), *LDMC* (g·g^−1^), *LWC* (g·g^−1^), and *LTD* (g·cm^−3^) were calculated as follows [49]:

*SLA* = *LA*/*LDW*

*LDMC* = *LDW*/*LTW*

*LWC* = (*LLFW* – *LDW*)/*LFW*

*LTD* = *LDW*/(*LA* × *LT*)

- Plant functional traits:

The *P_n_*, *T_r_*, *G_s_*, and *C_i_* of plants were measured in vivo using a Li–6400 portable photosynthesis system (LI–Cor Inc., Lincoln, NE, USA) [50]. We selected sunny weather and 9:00–11:00 a.m. every day for the dominant shrubs and herbs, and we selected fully expanded, healthy, mature, and sunny leaves as the measurement object. The open air path mode was used in the measurement. The air flow rate was set at 500 cm^3^·min^−1^, the atmospheric temperature was controlled at 26 ± 2 °C, the relative humidity of the air was maintained at 50–70%, the CO_2_ concentration was set at 400 ± 10 μmol·mol^−1^, and the light intensity was set at 1200 μmol·m^−2^·s^−1^ [51]. Each plant was measured every 1 h. Three leaves were selected for each measurement, and each leaf was measured three times.

#### 4.2.5. Sample Processing and Determination

The preparation and determination of samples were based on the industry standard LY/T 1210–1275–1999 [52]. The C, N, P, and K contents in the plant samples were determined by the potassium dichromate oxidation external heating method, the Kjeldahl method, the molybdenum antimony anti-colorimetric method, and the flame photometric method, respectively. The Ca and Mg contents were determined by atomic absorption spectrophotometry. Soil pH, SOC, total N, total P, and total K were determined by the potentiometric method, the potassium dichromate oxidation-external heating method, the semi-micro Kjeldahl method, the alkali fusion–molybdenum antimony anti-colorimetric method, and the alkali fusion–flame photometric method, respectively. Hydrolyzed N, available P, and available K were determined by the alkali hydrolysis–diffusion method, the hydrochloric acid–sulfuric acid extraction method, and the ammonium acetate extraction–flame photometric method, respectively. Total Ca and total Mg were determined by atomic absorption spectrophotometry, while exchangeable Ca and Mg were determined by ammonium acetate exchange–atomic absorption spectrophotometry.

#### 4.2.6. Data Processing and Analysis

In this study, the contents of C, N, P, K, Ca, and Mg in different components of shrubs and herbs were expressed by mass content, and the stoichiometric ratio was also the mass ratio. Excel 2016, SPSS 25.0, and Canoco 5.0 were used for data processing and statistical analysis. Origin 2021, Canoco 5.0, and Rstudio (2022.12.0+353) were used for data mapping. In order to ensure that the data meet the requirements of normal distribution and ANOVA hypothesis, the data were logarithmically converted (ln (x + 1)) before one-way ANOVA testing and correlation analysis. When performing post hoc multiple comparisons, Levene’s test is first used to test whether the variance is homogeneous. If the variance is homogeneous, the LSD method is used for multiple comparisons. If the variance is not homogeneous, Tamhane’s T2 method is used for multiple comparisons [53].

RDA was used to systematically analyze the effects of various ecological factors on the stoichiometric characteristics of karst forest plants [54]. In this study, the nutrient content and stoichiometric ratio of different components of shrubs and herbs were used as response variables, and topographic factors (altitude, slope, aspect, slope position, and microhabitat), soil properties (total N, hydrolyzable N, total P, available P, total K, available K, total Ca, exchangeable Ca, total Mg, exchangeable Mg, organic carbon, and pH value), and plant characteristics (plant species, ground diameter, and plant height (only shrubs) and *P_n_*, *G_s_*, *C_i_*, *T_r_*, *SLA*, *LDMC*, *LWC*, *LTD*) were selected as explanatory variables. Before constructing the RDA model, all numerical environmental variables are first standardized. Then, the collinearity diagnosis is performed with the help of the variance inflation factor (VIF), and the stepwise regression method is used to screen the variables. Finally, only the variables that contribute significantly to the interpretation of the model (*p* < 0.05) are retained into the constraint model. In order to evaluate the significance of the final constraint ranking model, 999 Monte Carlo permutation tests were used to further quantify the degree of explanation of each ecological factor with respect to the variation in the stoichiometric characteristics of different plant components.

In addition, in order to distinguish the independent and common interpretation contributions of different factor groups, VPA was carried out [55]. Based on the significant variables obtained from the above screening, the analysis was divided according to three prediction factor sets: the topographic factor set, the soil trait set, and the plant functional trait set. The varpart function of the vegan package in R 4.4.1 software was used to analyze the relative contribution of these three groups of variables and their interactions to the variation in the stoichiometric characteristics of different plant components. The goodness of fit of the model is evaluated according to adjusted *R*^2^ values to ensure the reliability of the explanatory power of the model. The final result is visualized by the plot function.

## 5. Conclusions

The C, N, P, K, Ca, and Mg contents and their stoichiometric ratios in the main shrubs and herbs in the karst area did not show significant differences at the microhabitat scale. However, in the same microhabitat, the contents of N, P, K, and Mg in shrub leaves were significantly higher than those in branches and roots, and the K content in herb leaves was also significantly higher than in roots. The C/N, C/P, and C/K of shrub branches and roots were significantly higher than those of leaves, and the C/K of herb roots was also significantly higher than that of leaves. This reflects the functional differentiation of plant components and the different trade-off strategies for resource acquisition and storage. There is a synergistic trade-off relationship between different components of plants, and the interaction between the element content and the stoichiometric ratio mainly manifests as antagonistic and synergistic effects. The stoichiometric characteristics of different shrubs and herb components are regulated by multiple factors, but their driving modes are significantly different. The stoichiometric characteristics of shrub leaves were mainly dominated by species characteristics and were synergistic with *P_n_* and slope position. The branches were affected by *T_r_* and soil-exchangeable Ca. Roots were dynamically regulated by altitude gradient and soil-exchangeable Ca. In contrast, the stoichiometric characteristics of herb leaves were dominated by *LTD*, while root characteristics were mainly affected by soil-exchangeable Ca. This difference is essentially a manifestation of the different adaptive strategies of two different plant forms to karst “high-Ca–low-P heterogeneous” habitats: shrubs directly obtain bedrock slow-release nutrients through deep roots penetrating rock gaps and combine high C/N and C/P to improve nutrient utilization efficiency, forming an adaptive “mechanical resistance priority–metabolic cost optimization” strategy; herbs, by contrast, respond to environmental fluctuations through the plasticity of functional traits, rely on shallow roots with high metabolic activity to secrete organic matter to activate surface soil P, and achieve light energy capture and rapid growth through high *SLA* and low *LTD*. In addition, plants also dynamically adjust the absorption ratio of elements (such as N/P and Ca/Mg) through the homeostasis mechanism to alleviate the stress of “high Ca–low P” in karst, which reflects the convergent adaptation mechanism of different plant forms to heterogeneous habitats. In this study, through the integration of niche scale analysis, multi-factor synergy analysis, and plant adaptation strategy discussion, the component specificity and environmental driving mechanism of the stoichiometric characteristics of karst shrubs and herbs were clarified, and the differences in the resource allocation strategies and niche differentiation of different plant forms were revealed, which provides a theoretical basis for understanding the stability maintenance of karst forest ecosystems and restoration practices for degraded karst forests.

## Figures and Tables

**Table 1:** Structural traits of the leaves of the main shrubs and herbs in karst forests.

Plant Forms	Microhabitats	*SLA* (cm^2^·g^−1^)	*LDMC* (g·g^−1^)	*LWC* (g·g^−1^)	*LTD* (g·cm^−3^)
Shrubs	Stone gully	166.70 ± 66.90	0.3775 ± 0.1159 A	0.5931 ± 0.1180	0.0495 ± 0.0216
	Stone surface	177.11 ± 65.66	0.3491 ± 0.1049 A	0.6119 ± 0.0945	0.0464 ± 0.0211
	Soil surface	180.03 ± 58.78	0.3343 ± 0.0928 A	0.6317 ± 0.0740	0.0451 ± 0.0155
Herbs	Stone gully	222.54 ± 131.92	0.2146 ± 0.0677 B	0.7533 ± 0.0952	0.0478 ± 0.0284
	Stone surface	294.70 ± 179.94	0.1985 ± 0.0783 B	0.7444 ± 0.1557	0.0350 ± 0.0224
	Soil surface	197.40 ± 99.95	0.2206 ± 0.0458 B	0.7170 ± 0.1046	0.0465 ± 0.0237

Different capital letters indicate significant differences among different plant forms in the same microhabitat (*p* < 0.05). The absence of any letter indicates that there is no significant difference between different microhabitats or different plant forms (*p* > 0.05).

**Table 2:** Functional traits of the leaves of the main shrubs and herbs in karst forests.

Plant Forms	Microhabitats	*P_n_*/(µmol·m^−2^·s^−1^)	*G_s_*/(mol·m^−2^·s^−1^)	*C_i_*/(µmol·mol^−1^)	*T_r_*/(mmol·m^−2^·s^−1^)
Shrubs	Stone gully	4.6421 ± 1.0079 A	0.0698 ± 0.0464	265.13 ± 60.45	0.7573 ± 0.1932
	Stone surface	4.8628 ± 1.3556	0.1149 ± 0.1242	276.77 ± 89.60	0.9143 ± 0.4548
	Soil surface	4.7126 ± 1.2368 A	0.0940 ± 0.0720	286.34 ± 74.36	0.8294 ± 0.2187
Herbs	Stone gully	2.5062 ± 1.1678 B	0.0599 ± 0.0691	258.14 ± 91.04	0.5350 ± 0.3006
	Stone surface	3.0908 ± 1.4858	0.1088 ± 0.0986	295.84 ± 101.43	0.8104 ± 0.4147
	Soil surface	2.3266 ± 1.8120 B	0.0596 ± 0.0792	273.94 ± 69.17	0.5603 ± 0.3270

Different capital letters indicate significant differences among different plant forms in the same microhabitat (*p* < 0.05). The absence of any letter indicates that there is no significant difference between different microhabitats or different plant forms (*p* > 0.05).

## Data Availability

The original contributions presented in this study are included in the article and Supplementary Materials. Further inquiries can be directed to the corresponding author.

## Supplementary Materials

The following supporting information can be downloaded at https://www.mdpi.com/article/10.3390/plants14182839/s1: Table S1: RDA ordination analysis of stoichiometric characteristics and environmental factors of main shrub plants in karst forests; Table S2: RDA ordination analysis of stoichiometric characteristics and environmental factors of the main herb plants in karst forests; Table S3: The significance test results and explanation degree of the effects of environmental factors on the stoichiometric characteristics of different components of the main shrubs and herbs in karst forests; Table S4: Basic overview of plant distribution table; Table S5: Microhabitat characteristics and soil chemical properties.

## Abbreviations

The following abbreviations are used in this manuscript:

*SLA*	Specific leaf area
*LDMC*	Leaf dry matter content
*LWC*	Leaf water content
*LTD*	Leaf tissue density
*P_n_*	Net photosynthetic rate
*T_r_*	Transpiration rate
*G**_s_*	Stomatal conductance
*C_i_*	Intercellular CO_2_ concentration
