# Supplementary material for: Influencing Factors and Adaptation Strategies of Stoichiometric Characteristics of Main Shrubs and Herbs in Karst Area at Microhabitat Scale
Source: Plants (Basel). 2025 Sep 11;14(18):2839. doi: 10.3390/plants14182839 (PMC12473311; doi:10.3390/plants14182839)
Supplement: Supplementary file 1 [file plants-14-02839-s001.zip › plants-3688531-supplementary.pdf]

Supplementary materials for

Influencing factors and adaptation strategies of stoichiometric characteristics of main shrubs and herbs in karst area at microhabitat scale

Tables S1. RDA ordination analysis of stoichiometric characteristics and environmental factors of main shrub plants in karst forests

Parameter	Leaves		Branches		Roots	
	Axis-1	Axis-2	Axis-1	Axis-2	Axis-1	Axis-2
Eigenvalues	0.4097	0.2935	0.3260	0.2470	0.3745	0.2302
Stoichiometry-environment correlations	0.9986	0.9996	0.9665	0.9666	0.9821	0.9937
Cumulative percentage variance of stoichiometry	40.97	70.32	32.60	57.30	37.45	60.47
Cumulative percentage variance of Stoichiometry -environment relation	41.29	70.87	34.99	61.50	38.73	62.54
F ratios of first canonical axis	1.3881		2.9021		2.3948	
p values of first canonical axis	0.0240		0.0340		0.0480	
F ratios of all canonical axes	14.0999		5.8464		7.2916	
p values of all canonical axes	0.0020		0.0020		0.0020	
Sum of all canonical eigenvalues	0.9922		0.9317		0.9669	
Sum of all eigenvalues	1.0000		1.0000		1.0000	

Tables S2. RDA ordination analysis of stoichiometric characteristics and environmental factors of main herb plants in karst forests

Parameter	Leaves		Roots	
	Axis-1	Axis-2	Axis-1	Axis-2
Eigenvalues	0.6642	0.1955	0.4201	0.3228
Stoichiometry-environment correlations	0.9985	0.9935	0.9939	0.9742
Cumulative percentage variance of stoichiometry	66.42	85.97	42.01	74.29
Cumulative percentage variance of Stoichiometry-environment relation	67.01	86.74	44.44	78.58
F ratios of first canonical axis	3.9561		2.1735	
p values of first canonical axis	0.0120		0.0140	
F ratios of all canonical axes	27.9893		7.4165	
p values of all canonical axes	0.0020		0.0020	
Sum of all canonical eigenvalues	0.9911		0.9454	
Sum of all eigenvalues	1.0000		1.0000	

Tables S3. The significance test results and explanation degree of the effects of environmental factors on the stoichiometric characteristics of different components of the main shrubs and herbs in karst forests

Life form	Plant components	Environmental factors	Explains (%)	F ratios	*p* values	Importance sequencing
Shrubs	Leaves	Plant species	34.3	9.9	0.002	1
		P_n	13.7	4.7	0.004	2
		Slope position	10.7	4.4	0.002	3
		Plant height	7.5	3.5	0.024	4
		Slope aspect	5.6	3.0	0.034	5
	Branches	T_r	19.4	4.6	0.002	1
		Soil exchangeable Ca	16.6	4.7	0.004	2
		Soil total Mg	8.7	2.7	0.038	3
	Roots	Elevation	18.2	4.2	0.008	1
		Soil exchangeable Ca	15.3	4.1	0.008	2
		Soil total Ca	14.0	6.4	0.004	3
		Slope aspect	12.9	4.1	0.010	4
Herbs	Leaves	*LTD*	61.5	14.4	0.002	1
		SLA	15.6	5.5	0.002	2
		Plant species	11.6	7.2	0.010	3
	Roots	Soil exchangeable Ca	38.7	5.7	0.004	1
		Plant species	17.6	3.2	0.034	2
		SLA	17.3	4.6	0.028	3

Tables S4. Basic overview of plant distribution table

NO.	Plant species	Life form	d (mm)	H (m)	Altitude (m)	Slope degree	Slope aspect	Slope position	Micro-habitat
1	*Nandina domestica* Thunb.	Shrub	10.9	1.2	719	15~25°	Semi-shady slope	Downslope	Stone gully
2	*Nandina domestica*	Shrub	16.0	1.9	704	15~25°	Shady slope	Downslope	Soil surface
3	*Nandina domestica*	Shrub	19.0	2.6	704	15~25°	Shady slope	Downslope	Stone surface
4	*Brassaiopsis glomerulata* (Blume) Regel.	Shrub	18.0	1.8	709	≤5°	Flat land	Depression	Stone surface
5	*Brassaiopsis glomerulata*	Shrub	25.0	2.5	709	≤5°	Flat land	Depression	Soil surface
6	*Brassaiopsis glomerulata*	Shrub	43.0	4.1	712	≥35°	Sunny slope	Downslope	Stone gully
7	*Miliusa sinensis* Finet & Gagnep.	Shrub	28.9	3.2	721	25~35°	Semi-shady slope	Downslope	Soil surface
8	*Miliusa sinensis*	Shrub	51.6	4.0	730	25~35°	Semi-shady slope	Downslope	Stone surface
9	*Miliusa sinensis*	Shrub	27.6	2.2	746	≥35°	Semi-sunny slope	Downslope	Stone gully
10	*Murraya exotica* L.	Shrub	32.7	3.0	728	≥35°	Semi-sunny slope	Downslope	Soil surface
11	*Murraya exotica*	Shrub	13.1	1.9	729	≥35°	Semi-sunny slope	Downslope	Stone surface
12	*Murraya exotica*	Shrub	32.5	2.5	707	≥35°	Semi-sunny slope	Downslope	Stone gully
13	*Chimonobambusa angustifolia* C. D. Chu & C. S. Chao.	Shrub	18.0	2.8	706	25~35°	Semi-sunny slope	Downslope	Stone gully
14	*Chimonobambusa angustifolia*	Shrub	7.0	1.4	707	25~35°	Semi-shady slope	Downslope	Stone surface
15	*Chimonobambusa angustifolia*	Shrub	9.0	1.7	704	5~15°	Semi-sunny slope	Downslope	Soil surface
16	*Indocalamus tessellatus* (Munro) P. C. Keng.	Shrub	8.0	1.3	747	≥35°	Sunny slope	Midslope	Stone surface
17	*Indocalamus tessellatus*	Shrub	3.0	1.3	747	≥35°	Semi-sunny slope	Midslope	Soil surface
18	*Indocalamus tessellatus*	Shrub	5.0	1.5	747	≥35°	Semi-sunny slope	Midslope	Stone gully
19	*Mahonia cardiophylla* T. S. Ying & Boufford.	Shrub	66.0	2.5	725	5~15°	Semi-sunny slope	Downslope	Soil surface
20	*Mahonia cardiophylla*	Shrub	16.0	1.5	725	5~15°	Semi-sunny slope	Downslope	Stone gully

21	*Mahonia cardiophylla*	Shrub	15.0	1.3	725	5~15°	Semi-sunny slope	Downslope	Stone surface
22	*Strobilanthes maolanensis* Blume.	Herb	/	/	709	15~25°	Semi-shady slope	Downslope	Stone gully
23	*Strobilanthes maolanensis*	Herb	/	/	709	15~25°	Semi-shady slope	Downslope	Soil surface
24	*Strobilanthes maolanensis*	Herb	/	/	747	≤5°	Flat land	Depression	Stone surface
25	*Pilea cavaleriei* H. Lév.	Herb	/	/	740	5~15°	Semi-shady slope	Downslope	Stone surface
26	*Cyperus rotundus* L.	Herb	/	/	725	15~25°	Semi-shady slope	Downslope	Stone surface
27	*Cyperus rotundus*	Herb	/	/	727	15~25°	Semi-shady slope	Downslope	Stone gully
28	*Cyperus rotundus*	Herb	/	/	727	15~25°	Semi-shady slope	Downslope	Soil surface
29	*Cyperus rotundus*	Herb	/	/	731	25~35°	Semi-sunny slope	Downslope	Soil surface
30	*Pilea cavaleriei*	Herb	/	/	728	25~35°	Sunny slope	Downslope	Soil surface
31	*Pilea cavaleriei*	Herb	/	/	728	25~35°	Sunny slope	Downslope	Stone surface
32	*Pilea cavaleriei*	Herb	/	/	728	25~35°	Sunny slope	Downslope	Stone gully

Table S5. Microhabitat characteristics and soil chemical properties

Life forms	Micro-habitats	TN ($g\cdot kg^{-1}$)	HN ($mg\cdot kg^{-1}$)	TP ($g\cdot kg^{-1}$)	AP ($mg\cdot kg^{-1}$)	TK ($g\cdot kg^{-1}$)	AK ($mg\cdot kg^{-1}$)	TCa ($g\cdot kg^{-1}$)	ExCa ($cmol\cdot kg^{-1}$)	TMg ($g\cdot kg^{-1}$)	ExMg ($cmol\cdot kg^{-1}$)	SOC ($g\cdot kg^{-1}$)	pH	Characteristics of microhabitat
Shrubs	Stone gully	11.95 ± 5.66ab	623.49 ± 358.39	1.02 ± 0.33	2.62 ± 2.29	12.69 ± 2.49	283.84 ± 145.58	23.73 ± 14.25b	9.77 ± 3.91	6.89 ± 1.86	3.69 ± 0.61	131.31 ± 75.28ab	7.06 ± 0.22	Stone gully refers to a rock dissolution ditch or erosion ditch. The main characteristics are that the proportion of bare rock is greater than 50%, the grooves are deeper than 30 cm, and the soil-covered area is less than 1 m^2. Generally, the soil layer in a stone gully is thick, and the water and fertilizer retention ability is good, so it is not easily affected by short-term drought. The habitat
	Stone surface	16.67 ± 4.80a	766.22 ± 181.73	1.33 ± 0.32	3.08 ± 1.90	11.53 ± 3.18	404.58 ± 186.93	41.70 ± 25.52a	12.40 ± 4.84	8.70 ± 3.05	4.69 ± 2.34	195.31 ± 74.90a	7.05 ± 0.09	Stone surfaces refer to areas where the bedrock is completely exposed, or the bare rock ratio is greater 50%. The rock surface is usually soil-free or covered only by a small area (less than 1 m^2) and no more than 20 cm thick. The ventilation condition of the stone surface is good, but the water dispersion is fast, the water and fertilizer retention ability is weak, temporary drought occurs easily, and the habitat conditions are extremely harsh.
	Soil surface	8.19 ± 3.64b	541.67 ± 204.34	1.10 ± 0.53	1.51 ± 0.60	14.09 ± 3.08	270.87 ± 226.50	19.69 ± 5.44b	9.07 ± 4.77	7.25 ± 1.97	3.78 ± 0.62	88.38 ± 43.87b	7.13 ± 0.18	Soil surface refers to the microhabitat with uniform soil cover, continuous soil-covered area greater than 1 m^2 and the soil level development is complete. The soil layer on the surface is thick, and the ability to retain water and fertilizer is usually closely related to the soil area and the thickness of the soil layer. The ventilation condition is good, and the water loss is slow, so the soil condition is optimal, but it may still be affected by short-term drought.
Herbs	Stone gully	12.50 ± 6.82	774.97 ± 314.64	1.15 ± 0.43	2.91 ± 1.50	12.63 ± 4.59	311.03 ± 124.64	21.33 ± 1.03	19.48 ± 25.29	5.68 ± 2.33	6.76 ± 4.12	128.46 ± 55.49	7.34 ± 0.28	/
	Stone surface	19.12 ± 5.92	1040.78 ± 211.14	1.52 ± 0.29	6.17 ± 5.35	11.14 ± 4.24	513.33 ± 284.25	36.35 ± 21.48	24.30 ± 20.33	6.06 ± 3.79	5.31 ± 2.49	216.76 ± 92.14	7.10 ± 0.15	/
	Soil surface	11.69 ± 4.52	785.1 ± 268.26	1.17 ± 0.40	2.51 ± 1.43	12.39 ± 3.76	255.24 ± 126.10	23.17 ± 8.50	14.13 ± 16.14	5.99 ± 2.01	6.15 ± 2.98	124.97 ± 44.02	7.10 ± 0.10	/

* Different lowercase letters indicate a significant difference among different microhabitats of the same life form ($p < 0.05$)
